# Factors associated with parental recognition of a child's overweight status - a cross sectional study

**DOI:** 10.1186/1471-2458-11-665

**Published:** 2011-08-24

**Authors:** Marja L Vanhala, Sirkka M Keinänen-Kiukaanniemi, Kaisu M Kaikkonen, Jaana H Laitinen, Raija I Korpelainen

**Affiliations:** 1 Department of Sports and Exercise Medicine, Deaconess Institute of Oulu, Kajaaninkatu 17, 90100 Oulu, Finland; 2 The Institute of Health Science, University of Oulu, Oulu, Finland; 3 Finnish Institute of Occupational Health, Oulu, Finland

**Keywords:** overweight status, children, recognition, parents

## Abstract

**Background:**

Very few studies have evaluated the association between a child's lifestyle factors and their parent's ability to recognise the overweight status of their offspring. The aim of this study was to analyze the factors associated with a parent's ability to recognise their own offspring's overweight status.

**Methods:**

125 overweight children out of all 1,278 school beginners in Northern Finland were enrolled.

Weight and height were measured in health care clinics. Overweight status was defined by BMI according to internationally accepted criteria. A questionnaire to be filled in by parents was delivered by the school nurses. The parents were asked to evaluate their offspring's weight status. The child's eating habits and physical activity patterns were also enquired about. Factor groups of food and physical activity habits were formed by factor analysis. Binary logistic regression was performed using all variables associated with recognition of overweight status in univariate analyses. The significant risk factors in the final model are reported using odds ratios (ORs) and their 95% confidence intervals (CIs).

**Results:**

Fifty-seven percent (69/120) of the parents of the overweight children considered their child as normal weight. Child's BMI was positively associated with parental recognition of overweight (OR 3.59, CI 1.8 to 7.0). Overweight boys were less likely to be recognised than overweight girls (OR 0.14, CI 0.033 to 0.58). Child's healthy diet (OR 0.22, CI 0.091 to 0.54) and high physical activity (OR 0.29, CI 0.11 to 0.79) were inversely related to parental recognition of overweight status.

**Conclusions:**

Child's healthy eating habits and physical activity are inversely related to parental recognition of their offspring's overweight. These should be taken into account when planning prevention and treatment strategies for childhood obesity.

## Background

Parents who do not recognise the weight status of their own overweight children may be less likely to provide them support in order to achieve a healthy weight. However, a large proportion of parents do not recognise that their offspring is in fact overweight or obese [1-8]. According to a recent review less than 50% of parents successfully recognised their offspring's overweight status [1]. Parental recognition of child's overweight is important in order to implement prevention and treatment strategies early in life.

In previous studies involving a broad age range of children, parents of older children have been more likely to identify their child as overweight than parents of younger children [3,9]. In many studies, mothers have been more likely to identify their daughters as being overweight than their sons [9-13], but several studies have supported parental perception is not associated with the a child's gender [3,14-20]. Maternal weight status or a mother's awareness of her own overweight status seems not to be associated with awareness of her offspring's weight status [10,12,13,16,21]. However, mothers with lower BMI have been suggested to report their child as overweight more often than mother's with high BMI [9] but contradictory evidence also exits [19]. Previous studies have also revealed the positive relationship between maternal education and the ability to recognise a child's overweight status [13,15,22].

Few studies have evaluated the association between child's diet, physical activity and parent's ability to recognise their offspring's overweight status [3,21-23]. Small qualitative studies in pre-school aged children has reported findings that a child who has a healthy diet, is happy, active, and can accomplish normal childhood activities was not considered as being overweight, regardless of the child's BMI [21,23]. In a study by Eckstein, parents of overweight children were more likely to be concerned if they recognised their child as being inactive [3]. In opposition to these findings, Manios et al found that the likelihood of mothers' underestimation of their preschool aged child's weight status was significantly higher in children engaging in physical activity for less than 3 h/week [22].

The aim of the current study is to evaluate the association between sosiodemographic and lifestyle factors and parents' ability to recognise overweight status in their own seven year old children in Finland.

## Methods

### Subjects

In this cross sectional study all school beginners (born in 1997, n = 1278) in the city of Oulu were invited to participate in the study by school nurses in November 2004. The children were given an anonymous questionnaire to be filled in by their parents. Parents were informed about the objectives of the work and the voluntary nature of their and their children's participation in the survey. The research carried out was in compliance with the Helsinki Declaration. The study was approved by the Ethical Committee of Oulu University Hospital.

The parents were asked to collect and record their child's weight history from birth to current age from the health records. Weight (in underwear) and height (without shoes) was subsequently measured by a qualified nurse in health care clinic. Overweight status was defined as a body mass index (BMI) ≥ 85th centile, and obesity as a BMI ≥ 95th centile relative to reference data for the international population [24]. BMI was calculated as weight in kilograms divided by the square of height in meters (kg/m^2^).

### The outcome: parent's perception of her offspring's weight status

The parents were asked to rate their child's level of weight status using the statements: 'my child is underweight', 'normal weight', 'slightly overweight' and 'heavily overweight'. The parents classified their child as "underweight or normal weight" (BMI < 85^th ^centile), slightly or heavily overweight (BMI > = 95^th ^centile) or heavily overweight (BMI > = 95^th ^centile). These categories were finally defined as "normal", "overweight" and "obese". The parents' ratings were compared to the child's correct weight status.

### Information on the family

Information on the parents' marital status, educational level, age and lifestyle factors such as smoking were asked. Occurrences of certain chronic diseases like diabetes, heart diseases, asthma and rheumatoid disease in the family were also asked. The parents' own weight and height were asked, as well as the parents' perception of their own weight status. The parent's overweight was defined as a BMI ≥ 25 kg/m^2^.

### Physical activity and dietary patterns

Physical activity was assessed via a series of questions adapted from an Activity Questionnaire [25]. The number of hours spent in physical and sedentary activities during a usual week (playing, exercise, watching television, working on a computer, playing video games and reading) was asked. The parents were also asked to report the frequency of exercise sessions they had together with their child using the statements: 'seldom or never', 'sometimes', 'often'. Parents' perception of their children's physical activity level was asked: 'In your opinion, is this child as physical active as she/he should be?' The alternatives were 'no, she/he is not', 'yes, she/he is' and 'she/he is too active'.

There were questions about meal patterns, eating habits and food frequencies. The food frequency questionnaire has been developed by the National Public Health Institute and has been used since 1978 [26]. The questionnaire consisted of twenty-two questions on the inclusion of the typical foods eaten by Finnish children (for example porridge or muesli, cereals, pizza, hamburgers, milk, milk chocolate, yoghurt or pudding, cheese, sausages, ham, cookies and cakes, bun, berries, fruit, fresh vegetables, chocolate, candies, juice, light soft drink, sugared soft drink, chips). The food consumption frequencies were: many times per day, daily or almost daily, 3 to 5 times per week, 1 to 2 times per week and less than two times per week. A child's overeating was assessed by the question 'How often do you estimate that your child overeats?' using the statements: 'Never', 'Once a week', 'A few times per week' and 'Many times per day'. A child's appetite was also considered with the following: 'Which one of the next alternatives best describes your child's appetite?' The alternatives were 'very good appetite', 'good appetite', moderate appetite', 'poor appetite' and 'very poor appetite'.

### Statistical analysis

The data gathered was analysed with SPSS for Windows software, version 15.0.1. The analyses were performed using all available data without replacement of missing values. The association between explanatory factors and the recognition of a child's overweight status was first analysed using cross-tabulation and χ 2-test for the dichotomous variables and independent t-test for continuous variables.

Factor groups of food and physical activity habits were formed by factor analysis. When lifestyle factors were formed, the numbers of factors to be retained for the description of lifestyle patterns were determined by components with eigenvalue > 1 and examination of the scree plots. Eating and physical activity patterns were identified by principal component with varimax (orthogonal) rotation to identify non-correlated factors. Items with factor loadings ≥ 0.3 were used to calculate factor scores for each of the factors.

To assess how well the various background and lifestyle factors were able to explain the parents' recognition of a child's overweight status, multivariate logistic regression with enter method analysis was performed using all variables associated with the recognition of overweight status in univariate analyses. The significant risk factors in the final model are reported using odds ratios (ORs) and their 95% confidence intervals (CIs). Statistical significance was achieved when p < 0.05.

## Results

Of the 1278 children invited to participate, 855 (77%) returned the completed questionnaire. Eighty-five percent of questionnaires were filled by mothers, 12% by fathers and one percent by another caregiver or guardian. Measures for height and weight at seven years of age were available for 749 (59%) of the children (382 boys, 367 girls).

17% (125/749) of the children were classified overweight or obese according to the international criteria, while 8% (58/728) of the children were classified as overweight and 0.3% obese (2/728) by the parents. Over half (69/120) of the parents of the overweight (including obese) children and 18% (6/33) of the parents of obese children considered their children as normal weight. A small number of parents (9/607) believed their normal weight children were overweight. The parents of overweight daughters (n = 63) were more likely to accurately identify the weight status of their child than the parents of overweight sons (n = 57) (51% vs. 33%, p = 0.053).

A third (219/717) of the mothers and over half (352/645) of the fathers were classified as being overweight or obese (BMI ≥ 25). Of the overweight and obese mothers 8% (18/219) considered themselves as being of normal weight as did 45% (158/352) of the overweight and obese fathers. 13% (63/498) of the normal weight mothers and 3% (9/293) of the normal weight fathers considered themselves as overweight.

### Factors associated with the recognition of a child's overweight status

The parents' ability to recognize their child's overweight status was positively associated with the child's BMI, mother's age and father's body mass index. There was a negative relationship between the child's age and correct rating (table 1). The accuracy of the parents' perception of their child's weight status was not associated with the accuracy of perception on the parents' own weight status. The children, whose overweight status was recognised by their parents, had less close relatives with heart disease (table 1). A married parent was less likely to recognise her child's overweight status than a parent who was divorced, widowed or unmarried (table 1).

**Table 1:** Characteristics of the 125 overweight and obese children and their parents by the accuracy of the parental perception of the child's overweight status.

	All overweight and obese children	Offspring's overweight status		
	n = 125	Is recognised n = 51	Not recognised n = 69	P ^1^
Mean age of the child, years (SD)	7.3 (0.3)	7.2 (0.3)	7.4 (0.3)	.024
Mean BMI of the child, kg/m^2 ^(SD)	19.9 (2.0)	21.2 (2.5)	18.9 (1.0)	< .001
Mean age of the father, years (SD)	39.0 (5.4)	40.1 (5.8)	38.3 (5.0)	.090
Mean age of the mother, years (SD)	36.9 (5.1)	38.1 (4.3)	26.2 (5.6)	.044
Mean BMI of the mother, kg/m^2 ^(SD)	25.2 (4.2)	25.8 (4.7)	24.7 (3.9)	.155
Mean BMI of the father, kg/m^2 ^(SD)	27.2 (3.5)	27.5 (2.9)	27.0 (4.0)	.472
Girls n (%)	66/125 (52.8)	32/51 (62.7)	31/69 (44.9)	.053
Boys	59/125 (47.2)	19/51 (37.3)	38/69 (55.1)	
Overweight father (BMI ≥ 25) n (%)	74/107 (69.2)	36/43 (83.7)	35/61 (57.4)	.004
Normal weight father	33/107 (30.8)	7/43 (16.3)	26/61 (42.6)	
Overweight mother (BMI ≥ 25) n (%)	50/118 (42.4)	25/49 (51.0)	23/65 (35.4)	.094
Normal weight mother	68/118 (57.6)	24/49 (49.0)	42/65 (64.6)	
Mother classifies her weight status correctly n (%)	101/116 (87.1)	41/48 (85.4)	59/65 (90.8)	.378
Mother classify her weight status incorrectly	15/116 (12.9)	7/48 (14.6)	6/65 (9.2)	
Father classifies her weight status correctly n (%)	78/102 (76.5)	30/40 (75.0)	46/60 (76.7)	.848
Father classify her weight status incorrectly	24/102 (23.5)	10/40 (25.0)	14/60 (23.3)	
Parents' are married or cohabiting % n (%)	109/124 (87.2)	40/51 (78.4)	65/69 (94.2)	.010
Divorced, widowed, unmarried	15/124 (12.1)	11/51 (21.6)	4/69 (5.8)	
Heart disease in family % n (%)	84/120 (70.0)	30/50 (60.0)	51/66 (77.3)	.045
No heart disease in family	36/120 (30.0)	20/50 (40.0)	15/66 (22.7)	
Father's education high school or higher n (%)	57/117 (48.7)	22/47 (46.8)	33/66 (50.0)	.738
Lower than high school	60/117 (51.3)	25/47 (53.2)	33/66 (50.0)	
Mother's education high school or higher n (%)	85/123 (69.1)	35/51(68.6)	47/68 (69.1)	.954
Lower than high school	38/123 (30.9)	16/51 (31.4)	21/68 (30.9)	

Values are means (SD) for child and parent age and BMI, and n (percentages) of frequency data.

^1 ^Independent t-test for continuous variables and χ ^2 ^- test for frequency data.

### Lifestyle patterns associated with recognition of child's overweight status

The variables associated with the parents' perception of the child's overweight status in univariate analysis were 'How often does your child eat porridge or muesli?', 'How often does your child eat fruits?', 'How often does your child eat berries or food made of berries?', 'How often does your child eat too much?', How would you describe your child's appetite?', 'How often do parents exercise together with their child?' and 'How active is your child?' (table 2).

**Table 2:** Lifestyle factors and factor groups associated with the identification on child's overweight status in the univariate logistic regression analysis

	Univariate analysis		
	Odds Ratio	95% CI	P
A child eats porridge and muesli	0.687	0.487; 0.971	.033
A child eats berries	0.529	0.324; 0.864	.011
A child eats fruit	0.562	0.379; 0.833	.004
Factor 1. Healthy diet	0.537	0.338; 0.853	.008
A child eats fresh vegetables	1.272	0.913; 1.773	.155
A child eats ice cream	1.048	0.631; 1.741	.856
A child eats hamburger	1.076	0.476; 2.432	.860
A child eats pizza	1.036	0.392; 2.736	.944
A child eats candies	0.706	0.286; 1.745	.451
A child eats chips	1.125	0.373; 3.390	.834
A child has a good appetite	1.841	1.060; 3.199	.030
A child overeats	1.949	1.247; 3.049	.003
Factor 2. Eating too much	2.136	1.293; 3.529	.003
A child plays computer	0.869	0.497; 1.521	.624
A child watches television	1.274	0.728; 2.229	.396
A child exercise with parents	0.364	0.169; 0.781	.010
A child's is physically active	0.155	0.053; 0.451	.001
Factor 3. Physically active	0.333	0.177; 0.627	.001

Three lifestyle factor solutions were identified. Factor 1 showed a "Healthy diet" pattern with high levels of fruits, berries and porridge in the child's diet. An "Eating too much" pattern was seen in factor 2 (a child has good appetite and eats too much) whereas the third factor showed a "Physically active" pattern with an active child exercising frequently with her parents. Factor 1 was the dominant lifestyle pattern and explained 16.0% of the variance of the response variables, and the remaining factors explained 12.8% and 8.8% of the variance, respectively. Together, the three factors explained 37.6% of the variance.

'Healthy diet' and 'Physically active' factor scores were lower in the group 'child's overweight status is recognised by parent' than in the group 'child's overweight status is not recognised' (-0,27 (SD 0.78) vs. 0.19 (SD 0.96), p = 0.006 and -0,29 (SD 0.86) vs. 0.19 (SD 0.45), p = 0.001 respectively). 'Eating too much' factor scores were higher in the group 'child's overweight status is recognised by parent' than in group 'child's overweight status is not recognised' (0.25 (SD 0.81) vs. -0.22 (SD 0.78), p = 0.002).

### Factors associated with identification of a child's overweight status in multivariate analysis

The results of binary logistic regression analyses for the perception of child's weight status are presented in table 3. Child's BMI was positively associated with parental recognition of overweight. Overweight status was identified more frequently in girls than in boys. A child's healthy diet and high physical activity were inversely related with parental recognition of overweight status in multivariate analysis. The model explained 71% of the variance (Nagelkerke R square).

**Table 3:** Factors and factor groups associated with the identification on child's overweight status in the multivariate logistic regression analysis

	Multivariate analysis		
	Odds Ratio	95% CI	P
Child's BMI	3.586	1.835; 7.010	< .001
Gender (boy vs. girl)	0.138	0.033; 0.577	.007
Child's age	0.083	0.011; 0.645	.017
Parents' marital status (married vs. no married)	0.804	0.098; 6.618	.839
Heart diseases in family (no vs. yes)	3.902	0.987; 15.429	.052
Mother's age	1.125	0.976; 1.296	.104
Factor 1. Healthy diet	0.221	0.091; 0.539	.001
Factor 2. Eating too much	2.290	0.981; 5.342	.055
Factor 3. Physically active	0.290	0.106; 0.794	.016

## Discussion

In this cross-sectional study, more than half of the parents' with an overweight or obese seven year old child did not recognise their child's overweight status. Daughters' overweight status was identified better than sons, and physically active children with healthy eating habits had lower probability for parental identification of overweight status.

The low rate of overweight recognition in our study is comparable with previous studies [1-8]. It seems that overweight children must be obese in order to be recognized as overweight [19]. Also in our study, the higher the child's BMI was, the better the overweight was identified, although even 18% of the parents of obese children considered their children as normal weight. It is not fully understood why parents so easily mistake their child's weight as being normal in cases where the child is overweight. Jain et al. reported that overweight children were described as "thick" or "big boned" by their mainly overweight mothers [21]. In a study of Adams et al, Native-American big children were viewed more positively as "cute and healthy," whereas "skinny" (i.e., normal weight) children were viewed more negatively as "underfed and sickly" [19]. Also they reported that some parents had experienced social pressure to "fatten up" their babies. We had no data on possible social factors underlying poor recognition of child's overweight for which an explanation remains to be found in future studies.

Children with healthy diet or high physical activity were classified as overweight less frequently by their parents than physically inactive children or children with poor eating habits. Our results are in agreement with some previous studies [3,21,23] but opposite findings also exist [22]. Manios et al found that mothers whose children were more physically active were less likely to underestimate their child's weight status [22]. Comparison of studies is difficult because of the varying age and ethnic and social background of the study populations and differences in methods used. 'Eating too much'-factor and heart diseases in the family were also associated with the perception of child's overweight status. However, the associations were not statistically significant.

We did not ask if the parents who recognised their child's overweight status were worried about their child's weight. According to the previous studies, parents are not always worried about their offspring's weight or health despite the offspring's overweight status [15,17-19,27], especially if a child has a healthy appetite and eats healthy foods, such as fruits and vegetables [21]. Should parents be aware and worried of a child's overweight status, if the child is physically active and has healthy eating habits? According to a recent review, the risks of obesity is greater than those from being sedentary, i.e., high physical activity reduces, but does not totally offset risks related to obesity [28]. The parental awareness of a child's obesity should be promoted, remembering at the same time, physical activity is highly recommended and should be encouraged, especially in overweight and obese children [29].

In opposition to the previous findings which revealed that mothers with lower educational levels are more likely to misclassify their children's weight status [13,15,22], we did not find any association between parental education level and the recognition of childhood overweight status. In our study, about 70% of the mothers had high education which may have affected the results.

Like many other studies, we found that overweight girls were more frequently identified as overweight by their parent's than boys [9-13]. In our study, mothers recognised their own overweight status better compared to the fathers, which has also been observed in previous studies [30-32]. A relatively small proportion (8%) of overweight mothers considered themselves being normal weight compared to previous studies, where 20-40% of overweight women failed to perceive their weight as being too high [33,34]. This may be a result from high education level of mothers in our study. High educated women have been shown to consider themselves as overweight more often than low educated women [31,33]. Many studies have reported that women and adolescent girls more often try to lose weight than boys [35,36], and they are more likely than men to be dissatisfied with their body image and size [37], which reflect that Western culture particularly pressures women to be of normal weight or even slim. The fact that parents identify their daughters overweight status better than their sons may also reflect this cultural phenomenon.

This study does have some limitations. Questionnaires were predominantly filled in by mothers, and the classification of the child's weight status was mainly based upon their mother's impression. Physical activity levels were not measured objectively, and we had no information on energy intake or portion sizes. The use of accelerometer or food diaries would have provided more valid data. However, questionnaires are common, low-cost methods particularly in large epidemiological studies. Data on parental height and weight were based on self-reports. In our data, 30% of mothers were classified as overweight. According to previous reports, prevalence of overweight has been reported to be 55% in women aged 35-44 years in North-Finland [38]. Thus, the mothers in our study seem to have underestimated their weight.

## Conclusions

Most parents with an overweight or obese seven year old child seem not to recognise their child's overweight status, especially if the child is physically active and has healthy eating habits. This should be taken into account when planning interventions and educating health care personnel. Healthcare providers should promote awareness of childhood obesity among community.

## Competing interests

The authors declare that they have no competing interests.

## Authors' contributions

The authors' responsibilities were as follows--MLV, RIK, KMK, SMK-K, JHL: designed the study; MLV: analyzed the data and wrote the first draft of the manuscript; and all authors: provided advice and consultation and reviewed the final draft of the manuscript. All authors read and approved the final manuscript.

## Pre-publication history

The pre-publication history for this paper can be accessed here:

http://www.biomedcentral.com/1471-2458/11/665/prepub

## References

[B1] Parry LL Netuveli G Parry J Saxena S A systematic review of parental perception of overweight status in children J Ambul Care Manage 2008 31 253 268 18574384 10.1097/01.JAC.0000324671.29272.04

[B2] Chaimovitz R Issenman R Moffat T Persad R Body perception: do parents, their children, and their children's physicians perceive body image differently? J Pediatr Gastroenterol Nutr 2008 47 76 80 10.1097/MPG.0b013e31815a34 18607272

[B3] Eckstein KC Mikhail LM Ariza AJ Thomson JS Millard SC Binns HJ Parents' perceptions of their child's weight and health Pediatrics 2006 117 681 690 10.1542/peds.2005-0910 16510647

[B4] Hackie M Bowles CL Maternal perception of their overweight children Public Health Nurs 2007 24 538 546 10.1111/j.1525-1446.2007.00666.x 17973731

[B5] He M Evans A Are parents aware that their children are overweight or obese? Do they care? Can Fam Physician 2007 53 1493 1499 17872878 PMC2234629

[B6] Killion L Hughes SO Wendt JC Pease D Nicklas TA Minority mothers' perceptions of children's body size Int J Pediatr Obes 2006 1 96 102 10.1080/17477160600684286 17907321

[B7] Muhammad NA Omar K Shah SA Muthupalaniappen LA Arshad F Parental perception of their children's weight status, and its association with their nutrition and obesity knowledge Asia Pac J Clin Nutr 2008 17 597 602 19114396

[B8] Vuorela N Saha MT Salo MK Parents underestimate their child's overweight Acta Paediatr 2010 99 1374 1379 10.1111/j.1651-2227.2010.01829.x 20394589

[B9] Maynard LM Galuska DA Blanck HM Serdula MK Maternal perceptions of weight status of children Pediatrics 2003 111 1226 1231 12728143

[B10] Boutelle K Fulkerson JA Neumark-Sztainer D Story M Mothers' perceptions of their adolescents' weight status: are they accurate? Obes Res 2004 12 1754 1757 10.1038/oby.2004.217 15601969

[B11] Fisher L Fraser J Alexander C Caregivers' inability to identify childhood adiposity: a cross-sectional survey of rural children and their caregivers' attitudes Aust J Rural Health 2006 14 56 61 10.1111/j.1440-1584.2006.00764.x 16512790

[B12] Jeffery AN Voss LD Metcalf BS Alba S Wilkin TJ Parents' awareness of overweight in themselves and their children: cross sectional study within a cohort (EarlyBird 21) BMJ 2005 330 23 24 10.1136/bmj.38315.451539.F7 15567804 PMC539845

[B13] Baughcum AE Chamberlin LA Deeks CM Powers SW Whitaker RC Maternal perceptions of overweight preschool children Pediatrics 2000 106 1380 1386 10.1542/peds.106.6.1380 11099592

[B14] Carnell S Edwards C Croker H Boniface D Wardle J Parental perceptions of overweight in 3-5 y olds Int J Obes (Lond) 2005 29 353 355 10.1038/sj.ijo.0802889 15768040

[B15] Genovesi S Giussani M Faini A Vigorita F Pieruzzi F Strepparava MG Stella A Valsecchi MG Maternal perception of excess weight in children: a survey conducted by paediatricians in the province of Milan Acta Paediatr 2005 94 747 752 10.1080/08035250510028894 16188779

[B16] Jackson J Strauss CC Lee AA Hunter K Parents' accuracy in estimating child weight status Addict Behav 1990 15 65 68 10.1016/0306-4603(90)90007-K 2316412

[B17] Young-Hyman D Herman LJ Scott DL Schlundt DG Care giver perception of children's obesity-related health risk: a study of African American families Obes Res 2000 8 241 248 10.1038/oby.2000.28 10832767

[B18] Rich SS DiMarco NM Huettig C Essery EV Andersson E Sanborn CF Perceptions of health status and play activities in parents of overweight Hispanic toddlers and preschoolers Fam Community Health 2005 28 130 141 15778627 10.1097/00003727-200504000-00005

[B19] Adams AK Quinn RA Prince RJ Low recognition of childhood overweight and disease risk among Native-American caregivers Obes Res 2005 13 146 152 10.1038/oby.2005.19 15761174

[B20] Rhee KE De Lago CW rscott-Mills T Mehta SD Davis RK Factors associated with parental readiness to make changes for overweight children Pediatrics 2005 116 e94 101 10.1542/peds.2004-2479 15995022

[B21] Jain A Sherman SN Chamberlin LA Carter Y Powers SW Whitaker RC Why don't low-income mothers worry about their preschoolers being overweight? Pediatrics 2001 107 1138 1146 10.1542/peds.107.5.1138 11331699

[B22] Manios Y Kondaki K Kourlaba G Vasilopoulou E Grammatikaki E Maternal perceptions of their child's weight status: the GENESIS study Public Health Nutr 2009 12 1099 1105 10.1017/S1368980008004412 19105868

[B23] Reifsnider E Flores-Vela AR Beckman-Mendez D Nguyen H Keller C Dowdall-Smith S Perceptions of children's body sizes among mothers living on the Texas-Mexico border (La Frontera) Public Health Nurs 2006 23 488 495 10.1111/j.1525-1446.2006.00588.x 17096773

[B24] Cole TJ Bellizzi MC Flegal KM Dietz WH Establishing a standard definition for child overweight and obesity worldwide: international survey BMJ 2000 320 1240 1243 10.1136/bmj.320.7244.1240 10797032 PMC27365

[B25] Bar-Or O Children and Exercise in a clinical contest- an overview Pediatric Sports Medicine for the Practitioner 1983 New York: Springer-Verlag 66 87

[B26] Helakorpi S Berg M-A Uutela A Puska P Health Behaviour among Finnish Adult Population, Spring 1995 1995 Helsinki

[B27] Holm-Denoma JM Lewinsohn PM Gau JM Joiner TE Striegel-Moore R Otamendi A Parents' reports of the body shape and feeding habits of 36-month-old children: an investigation of gender differences Int J Eat Disord 2005 38 228 235 10.1002/eat.20180 16211630

[B28] Fogelholm M How physical activity can work? Int J Pediatr Obes 2008 3 Suppl 1 10 14 18278627 10.1080/17477160801896481

[B29] Brambilla P Pozzobon G Pietrobelli A Physical activity as the main therapeutic tool for metabolic syndrome in childhood Int J Obes (Lond) 2010 10.1038/ijo.2010.255 21139560

[B30] Bish CL Michels BH Maynard LM Serdula MK Thompson NJ Kettel KL Health-related quality of life and weight loss among overweight and obese U.S. adults, 2001 to 2002 Obesity (Silver Spring) 2006 14 2042 2053 10.1038/oby.2006.239 17135622

[B31] Chang VW Christakis NA Self-perception of weight appropriateness in the United States Am J Prev Med 2003 24 332 339 10.1016/S0749-3797(03)00020-5 12726871

[B32] Schieman S Pudrovska T Eccles R Perceptions of body weight among older adults: analyses of the intersection of gender, race, and socioeconomic status J Gerontol B Psychol Sci Soc Sci 2007 62 S415 S423 18079430 10.1093/geronb/62.6.s415

[B33] Alwan H Viswanathan B Williams J Paccaud F Bovet P Association between weight perception and socioeconomic status among adults in the Seychelles BMC Public Health 2010 10 467 10.1186/1471-2458-10-467 20696072 PMC2924291

[B34] Blokstra A Burns CM Seidell JC Perception of weight status and dieting behaviour in Dutch men and women Int J Obes Relat Metab Disord 1999 23 7 17 10.1038/sj.ijo.0800803 10094580

[B35] Weiss EC Galuska DA Khan LK Serdula MK Weight-control practices among U.S. adults, 2001-2002 Am J Prev Med 2006 31 18 24 10.1016/j.amepre.2006.03.016 16777538

[B36] Ojala K Vereecken C Valimaa R Currie C Villberg J Tynjala J Kannas L Attempts to lose weight among overweight and non-overweight adolescents: a cross-national survey Int J Behav Nutr Phys Act 2007 4 50 10.1186/1479-5868-4-50 17935629 PMC2174511

[B37] Strauss RS Self-reported weight status and dieting in a cross-sectional sample of young adolescents: National Health and Nutrition Examination Survey III Arch Pediatr Adolesc Med 1999 153 741 747 10401809 10.1001/archpedi.153.7.741

[B38] Helakorpi S Patja K Prättälä R Uutela A Health Behaviour and Health among the Finnish Adult Population, Spring 2006 2007

